# Unicompartmental knee replacement: controversies and technical considerations

**DOI:** 10.1186/s42836-024-00242-6

**Published:** 2024-05-02

**Authors:** Waleed Albishi, Nasser M. AbuDujain, Mohammed Aldhahri, Meshari Alzeer

**Affiliations:** 1 Department of Orthopedic Surgery, College of Medicine, King Saud University, Riyadh, 11362 Saudi Arabia; 2 Department of Family and Community Medicine, College of Medicine, King Saud University, Riyadh, 2925 Saudi Arabia; 3 College of Medicine, King Saud University, Riyadh, 11362 Saudi Arabia

**Keywords:** Unicompartmental knee replacement, Unicompartmental knee arthroplasty, Total knee replacement, Total knee arthroplasty, Cemented, Cementless, Mobile bearing, Fixed bearing, High tibial osteotomy, Return to sports

## Abstract

**Background:**

Unicompartmental knee replacement (UKR) is one of the effective interventions for the treatment of symptomatic knee osteoarthritis. Moreover, it has multiple advantages over total knee arthroplasty (TKA), including reduced intraoperative blood loss, decreased risk of transfusion, and faster recovery. This study aimed to discuss critical technical considerations regarding UKR and some of the controversies and updates.

**Methods:**

We conducted a review to provide an overview of the controversies and technical considerations about UKR in several aspects. Only peer-reviewed articles were included, up to December 2023 using PubMed, Google Scholar, ERIC, and Cochrane database for systematic reviews databases.

**Result:**

UKR is associated with superior patient-reported clinical and functional outcomes, as well as shorter hospital stays, fewer postoperative complications, and revealed favorable outcomes in patients’ return to sport. The choice between mobile- and fixed-bearing prostheses depends, in part, on the surgeon’s preference. The mobile-bearing UKR is a less constrained prosthesis and can potentially result in less wear, but it is more technically demanding. While no significant difference between mobile-bearing versus fixed-bearing prostheses, cementless is superior to cemented design. Furthermore, UKR can be a good alternative for high tibial osteotomy (HTO) and still can be considered after a failed HTO. Lastly, recent reviews have shown a revision rate comparable to that of TKA. This is probably influenced by Improved comprehension of the best indications, patient selection criteria, as well as of the design, materials, and technological advances.

**Conclusion:**

UKR treatment for unicompartmental knee osteoarthritis is secure and effective. Based on clinical and functional outcomes, decreased morbidity and mortality, and cost-effectiveness, long-term studies suggest that UKR is superior to TKA. Further investigation in this area is warranted.

## Introduction

Unicompartmental knee replacement (UKR) is a successful treatment for symptomatic end-stage osteoarthritis (OA) of the knee. Despite mounting evidence to the contrary, some surgeons still regard UKR as a specialized procedure for a small group of patients. Only 10% of orthopedic surgeons worldwide perform UKR. Given the apparent efficacy and safety of this minimally invasive method that could be administered to a wider majority of patients seeking knee replacement surgery, this figure is disappointingly low [1].

The criteria for selecting patients for UKR have been well described previously and have been expanded in recent years by the Oxford group [2]. It is impossible to overstate the necessity of strictly adhering to the guidelines for using this prosthesis, with accurate patient selection being a key factor in the success of this surgical procedure. An intact anterior cruciate ligament (ACL), bone-on-bone osteoarthrosis, and patients with a correctable deformity are among the related indications [2, 3].

UKR has multiple advantages over total knee arthroplasty (TKA), including reduced intraoperative blood loss, decreased risk of transfusion, and faster recovery. In addition, compared with TKA, UKR is associated with superior patient-reported clinical and functional outcomes, as well as shorter hospital stays, lower readmission rates, and fewer postoperative complications, such as thromboembolism, infection, stroke, and myocardial infarction [4]. This study aims to discuss important technical considerations regarding UKR (Table 1), as well as some of the controversies and updates.

**Table 1:** Summary of important technical considerations when performing UKR

Procedure	Technical considerations
Combined UKR with ACL reconstruction	• The ACL tibial tunnel is drilled slightly more lateral than usual and in a more vertical direction to avoid the risk of graft impingement on the prosthesis and to reduce medial stress on the proximal tibia • The tibial component is implanted on a slope not exceeding 7° • Ensure proper tensioning of the collateral ligaments and the ACL
UKR in patients with high BMI	• Preoperative full-length alignment radiographs should be obtained • A larger surgical incision compared to the classical UKR minimal surgical approach is performed to achieve adequate intraarticular visualization, protect the ACL, and achieve correct sizing and proper implantation of the prosthesis • The resection depth should be minimized to maximize the contact area between the implant and the strong subchondral bone • Adequate cortical bone support around the tibia baseplate during trialling and prior to final implantation is critical to avoid undersizing the prosthesis and minimize the risk of implant subsidence
Lateral UKR	• Lateral or medial parapatellar approach can be utilized • An additional transpatellar window can be added if needed, through which the saw plate can pass, to properly make the tibial cut • Tibial sagittal cut should be made in a slight internal rotation of approximately 10 to 15 degrees to compensate for the “screw-home mechanism”.^a^ • The femoral component should be placed in slight external rotation and as laterally as possible to avoid implant impingement on the tibial spine eminence as the knee moves from flexion to extension • Joint line elevation and overstuffing the lateral compartment should be avoided • The implant insert should be sized at knee full extension • Optimal alignment after lateral UKR should result in a slight undercorrection of the valgus deformity
Patellofemoral UKR	• Perform a careful arthrotomy to avoid injury to the tibiofemoral articular cartilage, menisci, or cruciate ligaments • The whiteside’s line and transepicondylar axis should be drawn as they will serve as references to judge the external rotation of the femoral anterior cutting guide.^b^ • Trochlear implant should be placed laterally without step off with the condylar cartilage and without impinging on the ACL • The patella should be prepared leaving at least 14 mm of bone thickness and using a component compatible with the future TKA when revision is required • The patellar button should be medialized, and the lateral osteophytes should be removed to prevent maltracking • Overstuffing the patellofemoral joint should be avoided • A lateral retinacular release can be performed in case of patellar maltracking or persistent patellar tilt

^a^To compensate for the “screw-home mechanism” as the knee moves from flexion to extension

^b^The presence of the “grand piano” sign following the anterior femoral cut is an indicator of a good external rotation

## Methods

We conducted a review as we were aiming to provide an overview of the Controversies and Technical Considerations about UKR in several aspects, such as UKR in ACL-deficient knees, in individuals with high BMI, in sport return, and some other specifications, given the enormous data available, rather than undertaking a scoping or systematic review. We only included peer-reviewed articles up to December 2023. Four databases were searched (PubMed, Google Scholar, ERIC, and Cochrane database for systematic reviews). Searching MeSH keywords included “Unicompartmental knee replacement”, “UKR”, “Unicompartmental knee arthroplasty”, “UKA”, “ACL”, “obese”, “high BMI”, “patellofemoral”, “sport”, “mobile-bearing”, “fixed-bearing”, “cement*”, “high tibial osteotomy”, and “bicompartmental”. Search has been attempted several times while alternating between the keywords as appropriate. In addition, reference lists of articles obtained from the search were also screened for possible additional relevant articles. All age groups, ethnicities, and genders were included in this review. Quality assessment of included studies was not undertaken; thus, we included all eligible articles.

## Discussion

### UKR in ACL-deficient knee

ACL insufficiency is frequently associated with symptomatic OA of the knee. Patients with end-stage medial compartment OA and ACL insufficiency should be aware of two separate pathologies when considering therapy choices. Secondary OA can occur in people with primary ACL deficits, which generally appear due to traumatic ACL rupture; these individuals are frequently young and active. Secondary ACL deficits, which are typically degenerative ACL ruptures, can emerge in patients with primary end-stage medial compartment OA; these individuals are frequently older [5].

According to popular opinion, a functionally-undamaged ACL is a necessary condition for UKR, and this procedure should not be performed in patients who have signs of ACL instability [6, 7]. A study examined whether UKR in ACL-deficient knees produces kinematics similar to typical UKRs in knees with intact ACLs. In the first 25% of a deep knee bend, the ACL-deficient group showed a large posterior femoral shift, but there was no variation in kinematic waveforms for any other activities except for enhanced medial AP translation in the ACL-deficient group during deep knee bend and stair descent [8]. UKR with ACL-deficient knees is associated with complications and revision rates of up to 21% higher at two years, mainly due to eccentric loading by posterior femoral subluxation, which leads to early tibial loosening [6, 7, 9, 10]. However, several studies have found UKR in patients with intact ACLs to have a 10–15-year survival rate of over 90%. The combination of UKR and ACL reconstruction has several advantages over TKA, such as less blood loss, better knee kinematics, bone stock preservation, and cost efficiency [11–13].

**Fig. 1:**
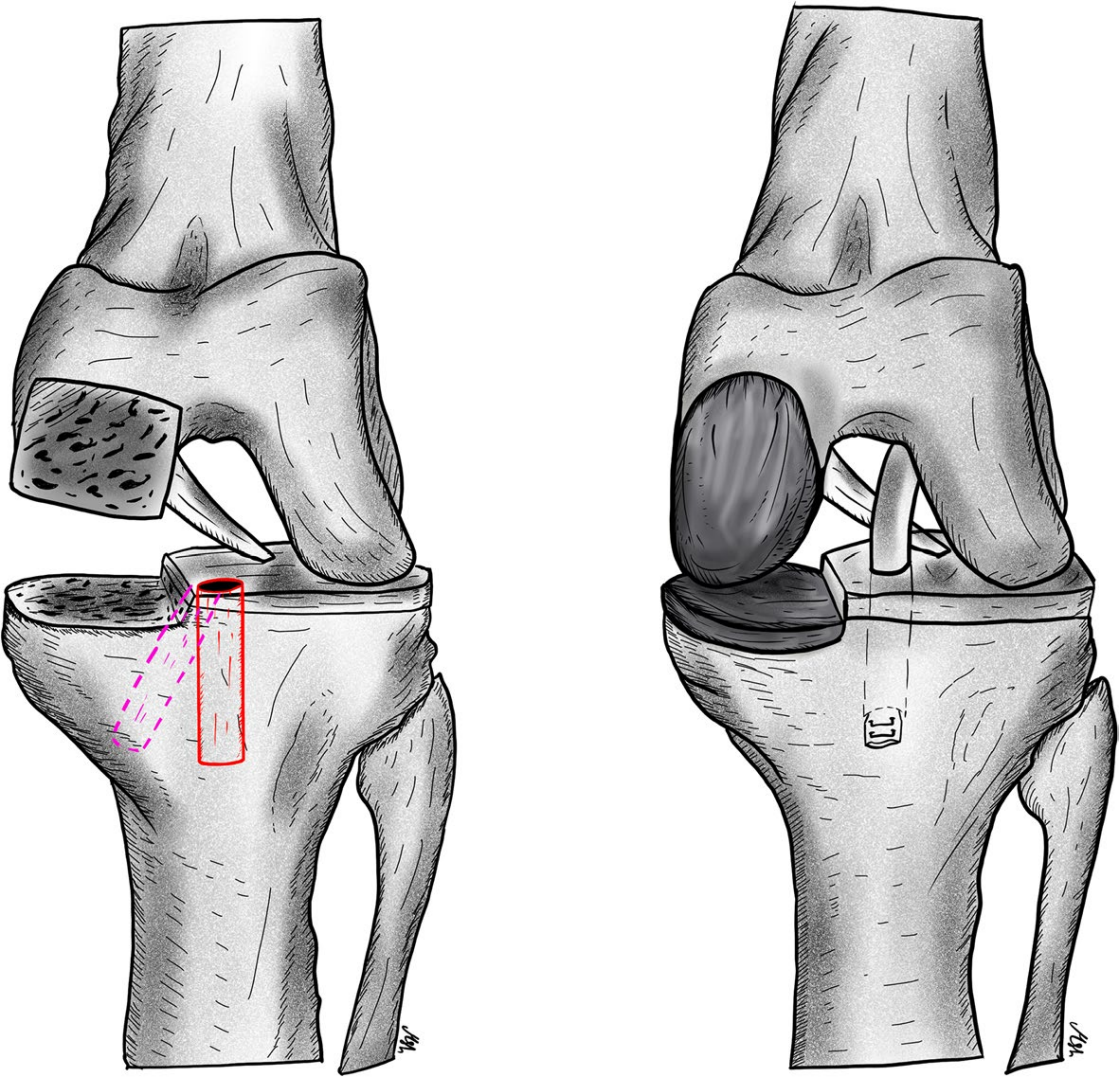
The ACL tibia tunnel is slightly lateralized and drilled in a more vertical orientation in the tibia to avoid graft impingement on the prosthesis and to reduce stress on the medial aspect of the tibial plateau

The optimal choice of graft is not clear in the literature. Hamstring and bone-patellar tendon-bone autografts are primarily used, but other options include the use of allografts and synthetic materials [5]. Technical factors, such as implanting a tibial component at a slope of no more than 7°, which potentially reduces the anterior translation of the tibia, will reduce the forces on the ACL [10]. In addition, proper tensioning of the collateral ligaments and the ACL is key for successful results [14]. Finally, making the tibial tunnel slightly more lateral than usual and positioning it in a more vertical direction can help avoid graft impingement on the tibial component and reduce medial stress on the proximal tibia, which can cause fracture (Fig. 1) [15–17].

Performing UKR with ACL reconstruction in one stage has multiple advantages, with no significant clinical differences compared to the two-stage approach. Without the need for two surgical procedures, shorter hospitalization and reduced cost are among the advantages. The drawbacks include a more technically demanding surgery with concerns of potential graft impingement, an undersized tibial baseplate, and postoperative stiffness [18, 19].

The choice between mobile- and fixed-bearing prostheses depends, in part, on the surgeon’s preference. The mobile-bearing UKR is a less constrained prosthesis and can potentially result in less wear and lower rates of loosening due to the inferior surface sliding over the tibial tray and the femoral component rolling on their superior aspect, but it is more technically demanding and includes the risk of inlay dislocation, especially when proper ligament tension is not achieved [5]. Midterm clinical and radiological outcomes have not shown a significant difference between the different prosthesis designs when combined with ACL reconstruction [20].

### UKR in patients with high BMI

Due to rising budget constraints, some healthcare organizations have established precise BMI cut-offs when considering patient eligibility for knee arthroplasty due to obesity-related complications. Obesity is a significant risk factor for OA, particularly in the knee joint [21, 22]. Evidence shows that the risk of knee OA increases with higher BMI [23]. Thirty trials with a total of 80,798 patients and an average follow-up period of 5.42 years were examined. According to the meta-analysis report, obesity did not result in worse postoperative outcomes after UKR; hence, UKR should not be regarded as contraindicated because of obesity [24].

Another study examined the impact of obesity on clinical outcomes and implant survivability in patients with a fixed-bearing UKR ten years after surgery. The control group consisted of 142 individuals with a preoperative BMI of less than 30 kg/m^2^, while the obese group consisted of 42 patients with a BMI of more than 30 kg/m^2^. Postoperatively, both groups showed significant advances in functional and quality-of-life assessments [25]. However, obesity was associated with a lower implant survival rate and a higher likelihood of revision after 10 years [25, 26]. Conversely, several studies that had long-term follow-ups concluded that obesity did not affect long-term results following UKR [27–30].

Regarding prosthesis design, some authors believe that mobile-bearing UKR disperses weight more efficiently, which may reduce the risk of loosening and lessen revision rates in obese patients [9, 31]. In addition, several authors have reported a lower implant survival rate when a fixed-bearing prosthesis was used in patients with a high BMI [32].

Revision due to the progression of OA in the contralateral compartment is consistent with the most common causes of revision described in the literature [32–34]. This complication can be accelerated by improper intraoperative techniques or poor patient selection. Obtaining preoperative standing long-leg radiographs is critical in obese patients, for whom a proper intraoperative alignment assessment can be difficult, as it is significantly affected by the patient’s obesity [35, 36]. Intriguingly, in a recent study, high BMI counts as a risk for increased revision rate, mainly in cemented-UKR with a level III evidence [37]. Overcorrection of a patient’s mechanical alignment by overstuffing the operated compartment is poorly tolerated and will require revision. Revision for aseptic loosening is not a common cause of failure in this patient population; ensuring optimal cortical bone support for the prosthesis and avoiding under-sizing of the tibial base plate can prevent this complication [32, 38, 39].

### Lateral UKR

Although the prevalence of isolated OA of the lateral compartment of the knee is between 5%–10%, the technical demands and the unfamiliarity with lateral UKR among surgeons lead to a decrease in the performance of this procedure [40, 41]. The lateral compartment has a unique anatomy and different biomechanics than the medial compartment; the lateral tibial condyle is convex, while the medial condyle is concave [42]. In addition, the lateral collateral ligament is loose in flexion, while the medial collateral ligament is tight. This laxity causes an increase in the lift-off of the femur condyle on the lateral side in flexion compared to the medial side (7 mm laterally and 2 mm medially) [43]. The “screw-home” mechanism is considered essential for knee stability when standing. External rotation of the tibia occurs between full extension and 20° of knee flexion, resulting in the two cruciate ligaments tightening, which positions the knee in maximum stability at the end of knee extension [43]. Despite these technical challenges, lateral UKR has had good clinical results and high patient satisfaction in multiple published series [44–47]. Fixed-bearing UKR survival has been reported to range from 92%–98% at 10 years [48]. Mobile-bearing prostheses have a 90%–98% survival rate at two years and a 90%–94% survival rate at three years [48]. Analysis of 2,052 lateral UKRs revealed a 93% survival rate with no discrepancies between fixed- and mobile-bearing implants [49]. Hariri et al., in their recent paper in 2023, reported fixed-bearing is superior to mobile-bearing as in mobile, there is a higher risk of dislocation, which results in failure [50]. Moreover, Fixed Lateral Oxford (FLO) UKR shows an excellent rate of survival at five years of treatment [51]. Compared to TKA, lateral UKR appears to have greater functional benefits in the treatment of isolated lateral OA [44]. According to Walker et al. [45], this procedure has helped relatively young patients return to sports; they found that 98% of these patients resumed their outdoor recreation, with two-thirds achieving optimal activity levels after surgery. Furthermore, the better the postoperative activity, the better the scores in the University of California Los Angeles activity scale (UCLA) [52]. Progression of OA in the contralateral compartment is the most frequently cited cause of failure following lateral UKR [53]. Unique to mobile-bearing implants, a 10% bearing dislocation rate has been reported, the majority of which occurred within the first year [43, 54, 55]. Although the cause of bearing dislocation following lateral mobile-bearing UKR is potentially multifactorial, joint line elevation is one of the most important avoidable causes. The joint line is elevated either by overmilling the distal femur in an attempt to match the flexion and extension gaps, or by overstuffing the lateral compartment by sizing the insert during flexion rather than full extension [56, 57]. The laxity of the lateral collateral ligament in flexion should be considered physiological; judging the balance in flexion can lead to mistakenly choosing a thicker tibial insert [48]. The component thickness for lateral UKR is considered appropriate when the gap between the tibia and femur is 2–3 mm with the application of varus stress at 0° of knee extension [58].

**Fig. 2:**
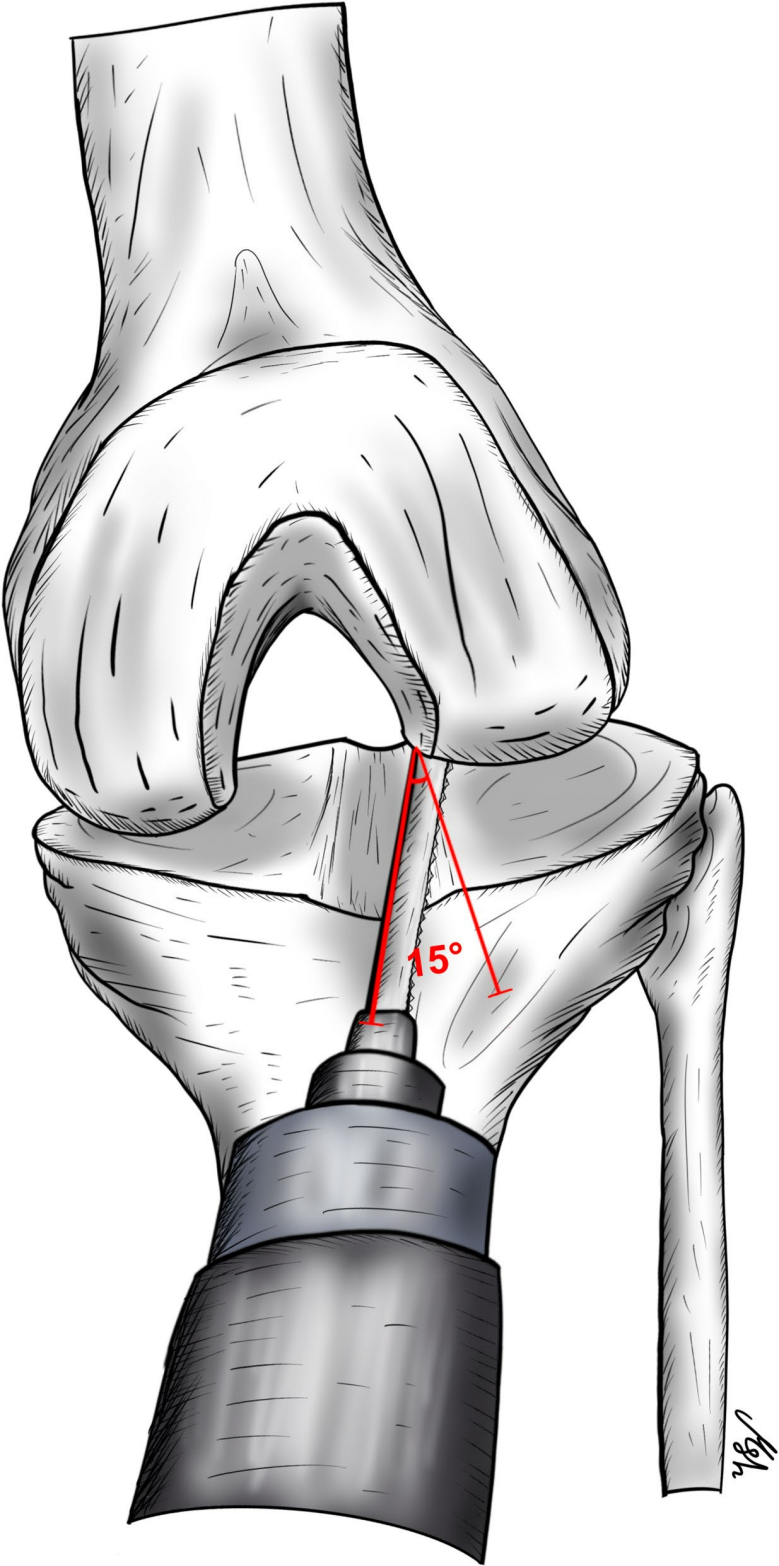
The sagittal cut for the tibia component of the lateral UKR is made in an internal rotation of 10 to 15 degrees to compensate for the “screw-home mechanism”

**Fig. 3:**
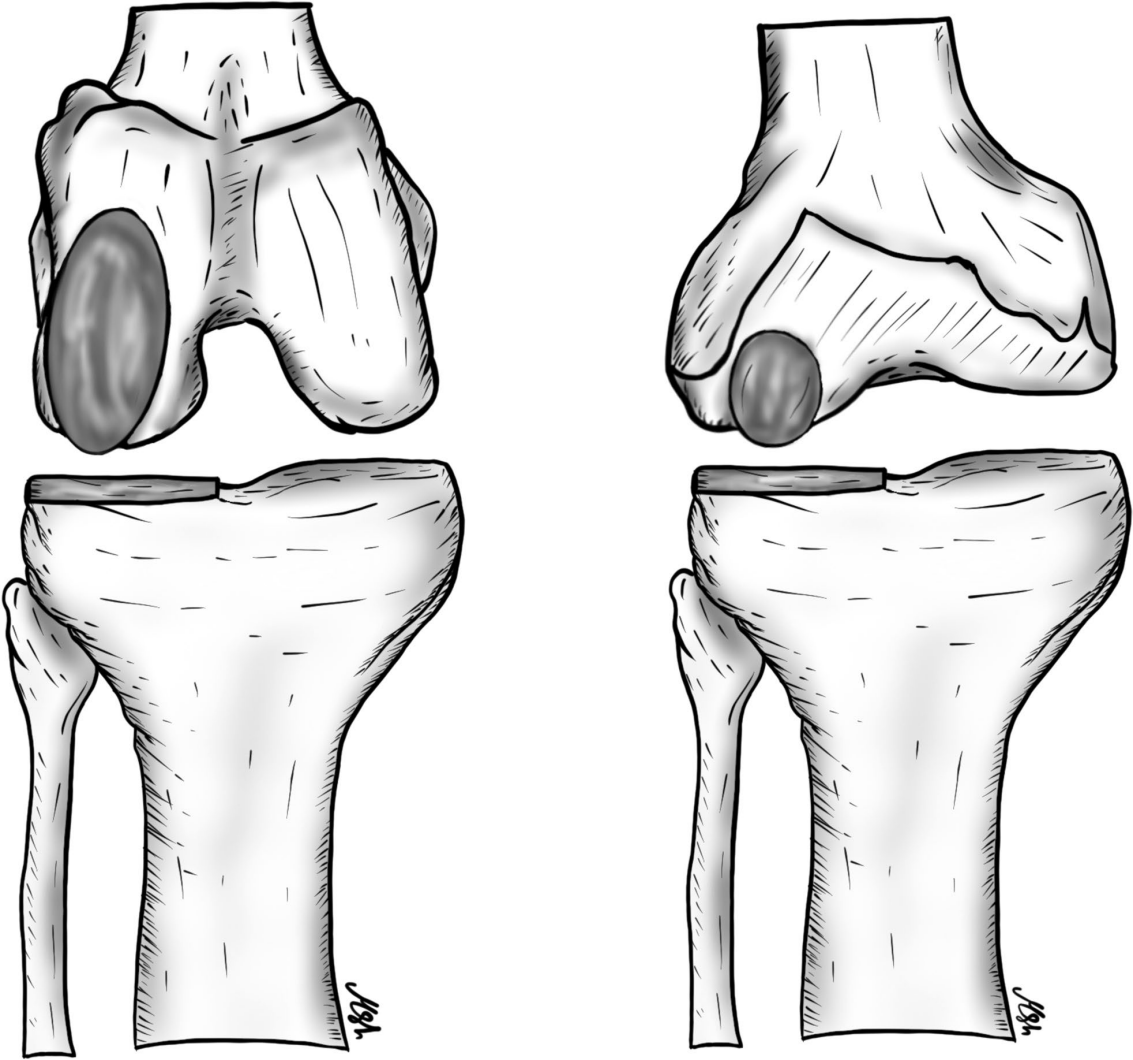
The UKR femoral component is positioned as laterally as possible and with slight external rotation to avoid implant impingement on the tibial spine eminence as the knee moves from flexion to extension

The tibial component should be placed along the lateral tibial spine to allow a larger area of the tibia to be covered while sitting on the rim of the cortical bone. Resection depth should also be minimized to maximize the contact area between the implant and the strong subchondral bone, thereby reducing the risk of implant subsidence into the weak metaphyseal bone [59, 48]. Furthermore, care should be taken to place the tibial component in a slight internal rotation (approximately 10°–15°) to compensate for the “screw-home mechanism” (Fig. 2) [48, 58]. In contrast, the femoral component should be placed in slight external rotation and positioned as laterally as possible to avoid implant impingement on the tibial spine eminence as the knee moves from flexion to extension (Fig. 3) [60].

Regarding the choice of surgical approach, lateral and medial parapatellar approaches have been described, both of which offer advantages and disadvantages. The medial approach is more extensile, with the ability to easily switch to TKA intraoperatively, but it requires a larger incision and includes the risk of damaging the medial structures. On the other hand, the lateral approach uses a small incision and provides direct visualization of the diseased lateral compartment without the need for patellar eversion [40, 48, 61]. The disadvantages include the difficulty of converting to TKA if the surgeon is unfamiliar with this approach and the tendency to externally rotate the tibial component if the patellar tendon is not properly retracted [62]. Thus, some authors have proposed making an additional trans-patellar incision through which the saw plate can pass to properly make an internally rotated tibial cut [63].

Controversies exist regarding optimal postoperative limb alignment. While over-correction can accelerate the development of OA in the nonoperative compartment, under-correction increases the load on the bearing surface and accelerates polyethylene wear [10, 13, 64, 65]. Optimal alignment after lateral UKR should result in a slight under-correction of the valgus deformity. Van der List et al. [66] reported improved functional scores in cases where 3°–7° of valgus was maintained compared with neutral alignment. A similar result was published by Donald W et al. when the postoperative femorotibial alignment was maintained at 5° valgus [58]. Although there is a lack of clinical studies examining the tibial component slope in lateral UKR, Chatellard et al. [67] reported higher failure rates when the tibial slope was less than 2° of the native slope. Similar findings were reported when the tibial slope was less than 2° or greater than 12° [68]. Donald W et al. [58] found excellent long-term results with an average posterior tibial slope of 6°.

In summary, lateral UKR provides excellent functional outcomes and great implant survival when the correct patient is selected and the technical aspects of the procedure are properly addressed [48]. Enhancements in lateral-specific implants and the redesign of the dome-shaped tibial component with the biconcave bearing lead to congruent contact between the components throughout flexion and increase the jump distance required for the bearing to dislocate [69]. These changes can translate into better operational efficiency and clinical outcomes.

### Patellofemoral arthroplasty

**Fig. 4:**
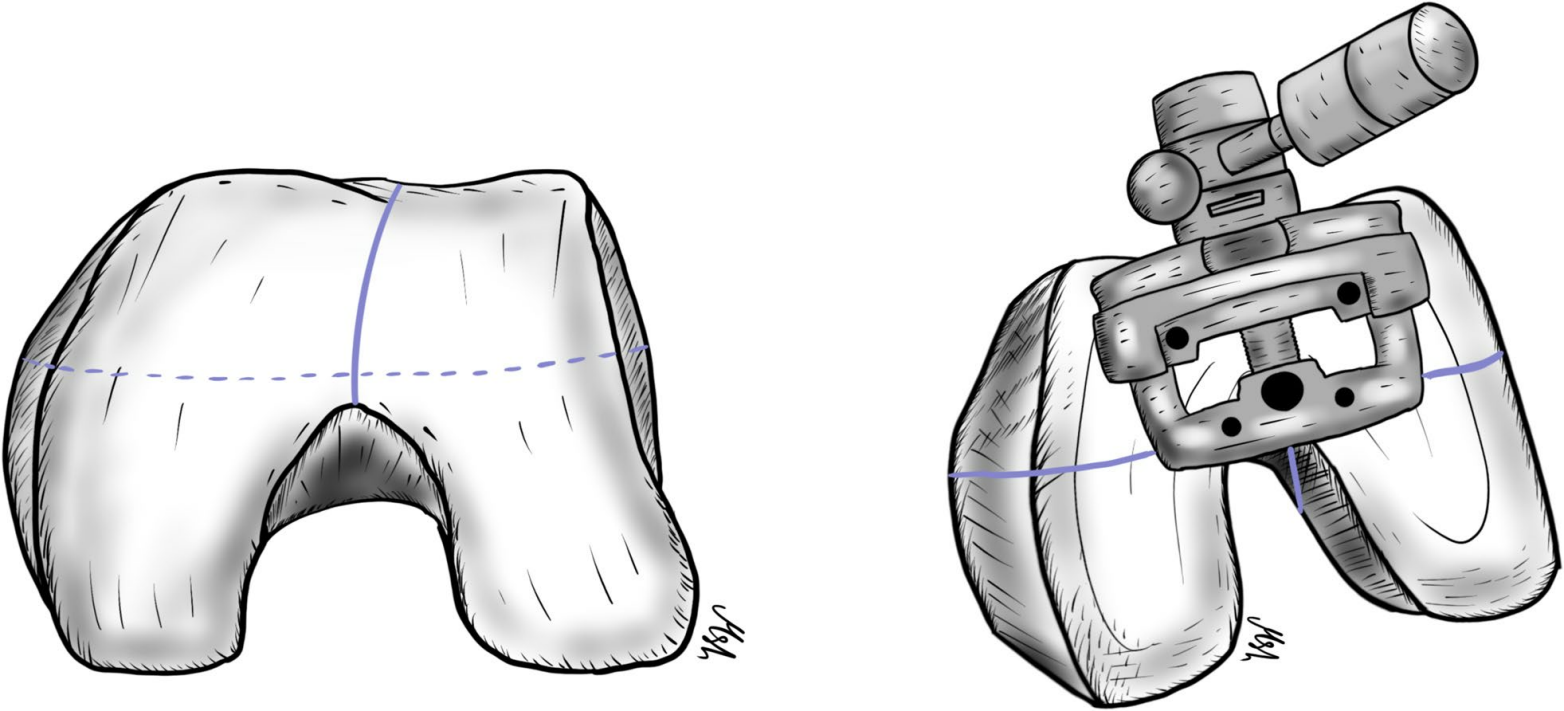
The whiteside’s line and Transepicondylar axis are drawn to serve as references to judge the external rotation of the femoral anterior cutting guide

**Fig. 5:**
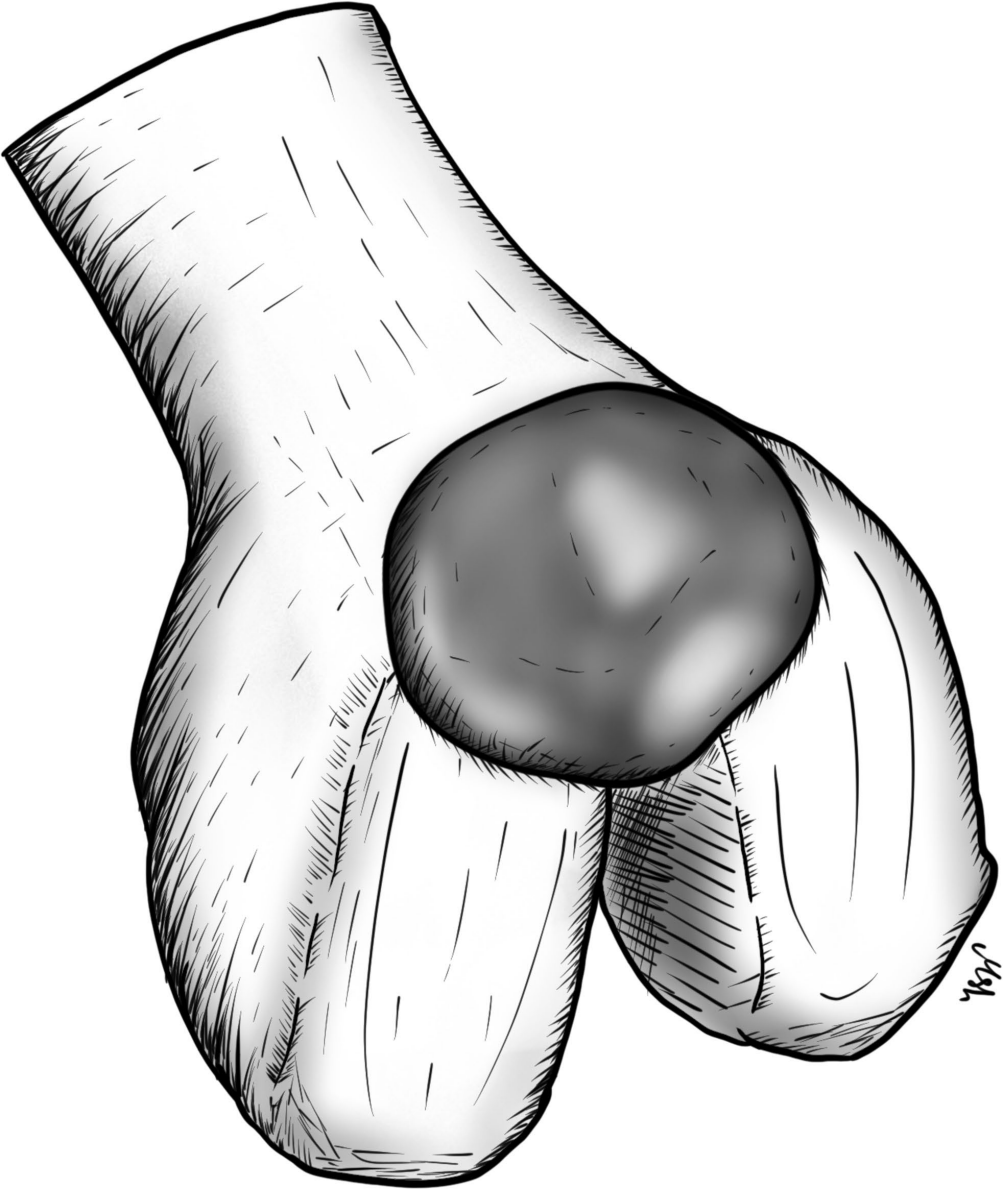
Trochlear component should be placed laterally without step-off with the condylar cartilage and without impinging on the ACL

The incidence of isolated patellofemoral (PF) OA ranges from 2%–11% in men and up to 24% in women [70, 71]. The higher incidence in females may be explained by the higher incidence of PF malalignment and dysplasia, which cause abnormal loading across the PF joint [72]. Patients with isolated PF OA usually complain of anterior or parapatellar knee pain that is aggravated by activities that increase the stress on the PF joint. These activities include going up or down stairs, squatting, kneeling, sitting for a long time while the knee is flexed, and standing from a seated position [71]. Non-surgical treatment options, including physiotherapy, knee braces, and injections, are usually offered to patients initially and may result in short-term improvement [73]. Surgical options range from simple debridement with or without offloading the tibia tubercle osteotomy to joint replacement [74]. Although TKA with patella resurfacing is a well-established procedure to treat this condition, anterior knee pain, which can persist in 19% of patients, as well as activity restrictions associated with this procedure, necessitate finding an alternative bone-preserving intervention that maintains joint kinematics, especially in young, active patients [71, 74, 75]. Compared to TKA, patellofemoral arthroplasty (PFA) preserves the tibiofemoral joint, the medial and lateral meniscus, and the cruciate ligaments, resulting in better postoperative knee function, higher activity scores, shorter hospital stays, and less blood loss [74, 76–79]. In addition, several meta-analyses have shown no significant differences in postoperative complications or revision rates between the two procedures. Several studies have shown that PFA can effectively delay the need for TKA by 10–15 years in 80% of patients [74, 79, 80]. The progression of OA in the other compartments of the knee is the most common cause of revision [66, 81]. Converting PFA to TKA is a classic and frequently utilized intervention, but some authors have proposed the addition of UKR in the diseased compartment, especially in young patients, to avoid revision and allow for the retention of the knee medial pivot, which consequently results in better knee kinematics [82, 83]. TKA can be performed without significant technical difficulty using a standard implant, and the clinical outcomes of TKA after failed PFA have been shown to be comparable to primary TKA and superior to revised TKA [74, 79, 80]. Although the use of the newer implant design (second generation only prosthesis) and the utilization of the correct surgical technique are important factors in the success of this procedure, proper patient selection is even more critical. Surgeons should avoid performing PFA in patients with tibiofemoral OA, as well as in those with uncorrectable tibiofemoral malalignment (more than 8° valgus or 5° varus deformity), knee instability, inflammatory joint disease, complex regional pain syndrome, and acute infection. The effect of obesity on PFA outcomes is controversial, with multiple conflicting data. Some authors have found lower patient satisfaction when a patient’s BMI is above 30, while other studies have shown that obesity does not affect outcomes [74, 79]. Regarding surgical technique, surgeons must keep in mind multiple technical considerations, including careful arthrotomy, to avoid injury to the tibiofemoral articular cartilage, menisci, or cruciate ligaments. A careful inspection of the entire knee compartment should be performed, and the diagnosis of isolated PF OA with intact ligaments should be confirmed before proceeding. Rotational alignment of the trochlear component is critical to avoid maltracking and to achieve good results. The white sideline and transepicondylar axis should be drawn. These marks serve as references to judge the external rotation of the femoral anterior cutting guide (Fig. 4). The presence of the “grand piano” sign following the anterior femoral cut is an indicator of good external rotation of the component. The final implant should be placed laterally without step-off with the condylar cartilage and without impinging on the ACL (Fig. 5). The patella should be prepared as in a standard TKA, leaving at least 14 mm of bone thickness and using a component compatible with a future TKA should revision be required. Another key point to prevent maltracking is the medialization of the patellar button and the removal of any existing lateral osteophytes. Finally, care should be taken to avoid overstuffing the PF joint; if patellar tracking is still inadequate or a patellar tilt is present, a lateral retinacular release should be performed [74]. In summary, with proper surgical techniques and careful patient selection, PFA is a bone-preserving procedure that has shown excellent clinical outcomes and nearly 90% survival in ten years [74]. It can be considered a bridging surgery for younger patients until they require conversion to TKA. Bond et al.’s recently reported that the new third-generation implants showed great promise, with outstanding functional outcomes and a significantly lower risk of mistracking and implant problems [84].

### UKR in young, active patients and return to sports

Return to sports (RTS) after unicompartmental knee replacement has been a concern of several studies. It is vital to address the expected consequences to meet both the patient’s expectations and the physician’s desired outcomes.

RTS after UKR has generally been found to be favorable, as in Kleeblad et al.’s cross-sectional study, which aimed to provide insight into patient satisfaction with RTS. The mean age of the participants was 62.3 years; after a follow-up of around two years, over 80% were satisfied with the restoration of their sports ability [85]. Similarly, Zimmerer et al. [86] and Walker et al. [87] evaluated RTS after UKR. Zimmerer et al. reported that 86% of the 19 patients surveyed were able to return to regular recreational and sporting activities [86], while Walker et al. revealed that the vast majority (98%) of the participants, independent of age or gender, returned to sports and recreational activity after lateral UKR with a general shift from high-impact to low-impact activities [87]. Radhakrishnan et al. [88] systematically reviewed time to RTS and the proportion of patients who returned to sports after UKR; their meta-analysis found that almost half the population was able to RTS at three months, and three-quarters were able to RTS at six months after UKR. In support of Walker et al.’s study [87], most of the patients who were able to RTS after UKR did so at a lower intensity than their preoperative level [88].

Some factors seem to affect the rapidity of RTS. Panzram et al. [89, 90] retrospectively reviewed what activity levels patients achieved, how they performed, and their quality of life with a well-functioning UKR, namely a cementless one. A fast return to activity with high levels of physical exercise was noted in most patients; however, those with an increased BMI tended to practice fewer types of sports and had decreased activity scores [89, 90].

Symptom improvement and good satisfaction levels have been shown after RTS in UKR patients. Walker et al. [91] found significant improvement in pain and knee function as well as 100% survivorship at a mean follow-up of two years. Plancher et al. [92] found that more than 80% of patients achieved an acceptable symptom state in terms of both daily life and sports activities. Regarding the latter, a good satisfaction level was reached in patients under 60 years old, who returned to satisfying and regular physical activities [93] with good function [94].

Proper postoperative rehabilitation can be an essential element of RTS. Dagneaux et al. [95] reviewed what factors influence RTS and concluded that an optimal range of movement and muscle-strengthening exercises facilitated a better return to activities that the patients practiced prior to the procedure [95]. The complexity of recuperation increases with more than one operation at a time. However, Kurien et al. [96] combined UKR with ACL reconstruction for patients presenting with isolated medial compartment OA and symptomatic ACL deficiency and found that a good RTS can be achieved when patients are chosen carefully [95].

### Mobile-bearing vs. fixed-bearing UKR

UKR was originally introduced as a fixed-bearing (FB) construct to address unicompartmental OA of the knee. More recently, mobile-bearing (MB) constructs have been introduced. Both methods have been studied individually and in comparison to each other; there remains discussion regarding which prosthetic design has superior long-term results [97].

MB UKR is a more congruent and less constrained prosthesis with a large contact area that disperses the load more efficiently, which may result in less wear and low rates of loosening due to the sliding motion of the inferior surface over the tibial tray and the rolling motion of the femoral component on their superior aspect, but it is more technically demanding, and without precise alignment and ligament balancing, it can lead to bearing dislocation or impingement [5]. On the other hand, an FB prosthesis often has a flat tibial articular surface, which is technically easier to implant, and there is no risk of bearing dislocation. However, an FB prosthesis is less conforming during flexion, which can lead to point loading and increase the risk of loosening. There are differences in the time and mode of failure between the two implant designs; bearing dislocation can lead to failures in MB prostheses, while FB prostheses can fail due to polyethylene wear [97].

Researchers have invested great effort in comparing MF and FB methods to reach a consensus on what might be in patients’ best interest. In terms of kinetics and function, Smith et al. [98] concluded that there were no significant differences in clinical outcomes between the two designs. Furthermore, in randomized controlled trials carried out by Ming et al. [99] and Confalonieri et al. [100], Ming et al. found a closer approximation of normal kinematics in the MB UKR; however, neither trial discovered benefits of one design over the other [99, 100]. In addition, no differences in range of motion or function were noticed between the designs at a two-year follow-up [101]. A 2013 meta-analysis on the same topic concluded that there were no significant differences between the two designs in terms of range of motion, limb alignment, and implant positioning [102]. These findings were further affirmed by a meta-analysis in 2022, where no advantages were found regarding using MB over FB implants for UKR in patients with monocompartmental knee OA [103]. In contrast, Hariri et al.’s paired analysis indicates that MB shows a superior range of motion than FB (FB: 118°, MB: 124°) [104]. Al-Rub et al. [105] and Neufeld et al. [106] addressed the survival rate of both MB and FB UKR designs; a similar overall survivorship between the two designs was found in both studies. Another comparison was made in terms of polyethylene wear, and the levels of wear-off and complications between the two designs were also assessed. Barrett et al.’s meta-analysis found that aseptic loosening occurs more frequently with FB implants and cemented fixation [107]. On the other hand, in Brockett et al.’s in vitro study, low-conforming FB UKR showed lower wear compared with the more conforming anterior–posterior sliding MB bearing [108].

### Cemented vs. cementless UKR

For several years, a cementless design has been opted for more frequently due to its advantages. Martin et al. [109] addressed the outcomes of cemented and cementless UKR in their cohort for five years after surgery; cementless UKR showed an association with better clinical outcomes than cemented one. The same claim was asserted by Manara et al. [110].

The revision rate has been shown to decrease with a cementless design. Three observational cohorts have described in the long-term follow-up (up to 10 years) that the cementless group showed a lower revision rate [111–113], while a systematic review of long-term revision rates in both cementless and cemented groups concluded that cementless UKRs offer equivalent, if not lower, revision rates to cemented UKRs [114]. Furthermore, regarding the extent of migration, Campi et al. [115] and Kendrick et al. [116] both reported no significant differences between the two components. In regard to pain, both cemented and cementless have profoundly decreased pain following UKR; however, cementless had significantly less pain than cemented UKR in all scores (Intermittent and Constant Osteoarthritis Pain [ICOAP], PainDETECT [PD], Charnley score, Oxford Knee Score [OKS] and American Knee Society Score [AKSS]) [117].

### UKR in patients who had high tibial osteotomy (HTO)

High tibial osteotomy (HTO) has always been regarded as an effective and extensively used technique for treating knee mal-alignment and unicompartmental arthrosis. TKA and HTO were the only options for treating unicompartmental arthrosis, and UKR has become increasingly popular [118]. UKR has been found to be a good alternative for HTO and is appropriate to perform after a failed HTO. For example, Yin et al. [119] investigated the clinical effectiveness of HTO compared to UKR in medial unicompartmental OA of knee patients, concluding that UKR can be a good alternative to HTO, as it can quickly restore the function of the knee joint. Furthermore, clinical and radiological assessments of UKR after a failed HTO conveyed the safety, effectiveness, and success of the procedure [120, 121]. Moreover, in a systematic review, Legnani et al. [122] reported on the outcomes of medial UKR after failed HTO, affirming the feasibility and satisfying outcomes of the procedure.

A recent large retrospective study by Bhattacharyya et al. included 96 patients to evaluate the survivorship of HTO in the treatment of medial compartment osteoarthritis. The survivorship at five postoperative years was 90.3%, and at ten years postoperatively, it was 82%, revealing a comparable survivorship between durations. Furthermore, a correlation existed between increasingly older age and more extensive adjustments needing a bone graft at the index procedure, which leads to a higher failure rate [123].

### Tibiofemoral UKR with coexisting patellofemoral knee OA

Knee OA is a common and disabling disease, and there are some cases where patellofemoral osteoarthritis (PFOA) and tibiofemoral osteoarthritis (TFOA) coexist. The impact of such coexistence has been explored in multiple research projects. Lu et al. [124], Deckard et al. [125], and Ercan et al. [126] concluded that whether coexisting OA is present does not significantly affect the outcome. Burger et al. [127] concluded that mild to moderate preoperative radiological degenerative changes, and mal-alignment of the PF joint are not associated with unfavorable outcomes. Similarly, Song et al. [128] and Hamilton et al. [129] agreed that mild or moderate OA of the PF does not stand as a contraindication for undergoing UKR.

### Bicompartmental knee arthroplasty

Bicompartmental knee replacement (BKR) is an emerging concept with several controversies. Romagnoli et al. [130] aimed to assess and describe the indications of bi- or unicompartmental and UKR combined with PF replacement; they addressed the trochlea anatomy related to morphotype, gender, and race. Accordingly, they concluded that using a customer-made replacement allows for selective replacement of the worn compartments and a customized fit of the small implants to the native knee anatomy, resulting in better overall function.

BKR has been compared with TKA on multiple occasions. After 48 months of follow-up, Confalonieri et al. [131] suggested that BKR maintains a high level of function and is a viable option for bicompartmental tibiofemoral arthritis, at least as good as TKA. Furthermore, it is associated with more favorable knee function and kinematics than TKA [132] and has equivalent survivorship but more remarkable improvement in functional outcomes in terms of revision [133].

On the other hand, a two-year follow-up comparison between UKR, BKR, and TKA by Al-Dadah et al. [134] indicated no significant differences among all groups. However, Elbardesy et al.’s meta-analysis [135] evaluated UKR versus TKA in the treatment of bicompartmental OA; a better short-term effect and a shorter operation time were associated with TKA compared with UKR. In the same line, the BKA group was found to have a higher chance of one- and two-year revision compared to UKR and TKA groups in Agarwal et al.’s recent retrospective cohort [136].

Monolithic off-the-shelf (OTS) and customized individually made (CIM) implants have been addressed in the context of UKR. Ogura et al. [137] evaluated the clinical outcomes after CIM-BKA with 55 participants and found that CIM-BKA allowed for a precise fit of the components and provided a significant improvement postoperatively, with a high level of satisfaction over short- to mid-term follow-up. Furthermore, Beckmann et al. [138] compared monolithic OTS-BKA with CIM-BKA, noting that CIM-BKA provides surgeons with a viable and patient-specific monolithic implant solution as an option for patients with bi-compartmental disease, and it compares favorably with revision rates for previously available monolithic OTS-BKA implants.

### UKR vs. total knee arthroplasty for knee OA

The utilization of UKR or TKA for the treatment of unicompartmental knee OA has been a subject of controversy among surgeons and in the literature, with differences in technique, rates of revision, and postoperative patient-reported outcomes. Mohammad HR et al. [139] and Hanna et al. [140] indicated that the functional and patient-reported outcomes post-UKR for the treatment of unicompartmental OA were significantly better from patients who underwent primary TKA across all age groups and demographics. Furthermore, Leiss et al. [141] concluded that patients across all age groups complained of less pain postoperatively after primary UKR and needed fewer pain-medications compared to primary TKA groups.

An important factor that must be considered when comparing UKR and TKA is functional outcome. Walker et al. [44] conducted a cross-sectional study that aimed to assess the functional outcomes of patients who underwent lateral UKR for the treatment of unicompartmental OA using the Oxford knee score (OKS) and range of motion (ROM). They compared the UKR patients’ results to those of patients who underwent TKA for the same indication; the UKR group showed more desirable results compared to the TKA group. Furthermore, Friesenbichler et al. [142] conducted a prospective study to assess isometric quadricep strength, spatio-temporal gait parameters (walking speed, step length, and single-limb support phase), and self-reported outcomes (pain, function, and stiffness) six months postoperatively in patients who underwent UKR for OA. They paired and compared their results with a TKA group and concluded that, after six months, UKR patients showed better quadricep strength and gait function compared to TKA patients, with less likelihood of stiffness and pain in the short term. Furthermore, Garner et al. [143] conducted a cross-sectional study in which they aimed to measure gait and patient-reported outcomes in the cases of partial knee arthroplasty and combined partial knee arthroplasty compared to TKA using the compartmental approach. They concluded that patients who underwent the compartmental approach reported better outcomes and had a more normal gait during follow-up compared to TKA patients.

UKR survivorship, revision, and reoperation rates have been debated in the literature. Hunt et al. [144] conducted a retrospective study in which they aimed to measure and compare the revision rate and the 90-day postoperative mortality rate of both UKR and TKA patients. Their results showed that the revision rate of UKR was significantly higher compared to TKA, even with different demographics and caseloads. Furthermore, the 90-day postoperative mortality rate was lower in UKR patients than in TKA patients. A similar study conducted by Kennedy et al. [145] showed that younger patients who underwent UKR had lower revision rates. Although the known rate of revision for UKR is higher than for primary TKA, some studies have indicated that mid-term survivorship and general patient outcomes are more prevalent in UKR cases [146].

The functional outcomes of UKR and TKA are a point of consideration for surgeons when choosing the proper primary procedure for patients being treated for knee OA, especially when comparing a revised UKR converted to a TKA, a revised TKA, and a primary TKA. Pearse et al. [147] conducted a study in which they aimed to measure the survivability and functional outcomes of a revised UKR to TKA and compared them to the outcomes of a primary TKA. They concluded that converting a failed UKR to a TKA showed significantly less favorable functional outcomes than a primary TKA in the short term, yet this was not the case when compared with revised TKA. Lombardi et al. [148] performed a retrospective study to measure postoperative outcomes in patients who underwent UKR to TKA revisions and compare the results with those of patients who underwent TKA revisions and primary TKAs. They deduced that the patients’ outcomes after UKR to TKA revisions were substantially better than TKA to TKA revisions and aligned more with primary TKA outcomes. Regarding the survivorship of a revised UKR or TKA, Masri et al. [149] showed that the reoperation and survival rates were similar in both UKR to TKA and TKA to TKA. In contrast to some of the literature, Jonas et al. [150] found that revised UKR to TKA cases had even better reported functional outcomes than primary TKA cases.

A recent study assessing the periprosthetic fracture rate between TKR and UKR showed that the fracture risk in both UKR and TKR was negligible, with minimal absolute variations across implant kinds. The fracture rate following UKR was 0.1% postoperatively in the first three months, which was roughly twice as high as the rate following TKR. However, in the first ten years following TKR, the cumulative fracture rate was 1%, nearly twice as high as following UKR. Patients 75 years of age or older, female patients, and patients with average body weight had increased fracture rates following both UKR and TKR [151].

According to the literature, surgeon caseload plays a role in defining the most probable outcome of a UKR. For example, Mohammed et al. [139] conducted a retrospective study in which one aim was to measure the revision and reoperation rates in surgeons with different caseloads. The results showed that UKR revision and reoperation rates were higher than those of TKAs in low-volume surgeons, but in mid-volume surgeons, the revision rate was similar, with an even higher reoperation rate. In high-volume surgeons, the data showed lower UKR reoperation rates, and revision rates similar to TKAs. Performing a UKR in non-inventor centers and inventor centers was also argued. R. Nandra et al. conducted a study that measured the five-year clinical outcomes of 257 patients post-Oxford UKR at a non-inventor center and found that the revision rate was lower than what is reported in the literature. Furthermore, the survival rate was similar to that of inventor centers, which supports the use of Oxford cementless UKRs outside of them. However, some surgeons still perform a TKA instead of a UKR for medial unicompartmental OA due to the learning curve related to the UKR procedure [152].

A limitation of our study is that we conducted a narrative review, not a systematic review, which can be lower on the evidence ladder; however, given the scope of the study and the abundance of relevant articles, a systematic review cannot cover it.

## Conclusion

The therapy of unicompartmental knee OA with UKR is both safe and efficacious. Long-term studies have implied that UKR is preferable to TKA based on clinical and functional outcomes, decreased morbidity and mortality, and cost-effectiveness. The revision rate in some studies was found to be higher; however, more recent reviews have shown a revision rate comparable to that of TKA. This is probably influenced by UKR’s propensity for revision, as well as inadequate patient selection criteria or surgical techniques. Improved comprehension of the best indications, patient selection criteria, as well as of the design, materials, and technological advances related thereto, could lower revision rates, and increase UKR survival. UKR is currently considered a viable option for most patients, regardless of age, activity level, or weight.

## Data Availability

Not applicable.
